# The toxic effects of *Helicobacter pylori* and benzo(a)pyrene in inducing atrophic gastritis and gut microbiota dysbiosis in Mongolian gerbils

**DOI:** 10.1002/fsn3.4368

**Published:** 2024-07-29

**Authors:** Yilun Huang, Yunxiang Chen, Lingfei Ma, Honggang Guo, Hao Chen, Bo Qiu, Mingfei Yao, Weixin Huang, Lian Zhu

**Affiliations:** ^1^ Alberta Institute, Wenzhou Medical University Wenzhou China; ^2^ Center for Safety Evaluation and Research Hangzhou Medical College Hangzhou China; ^3^ Institute for Health Policy Hangzhou Medical College Hangzhou China; ^4^ Center of Laboratory Animal Hangzhou Medical College Hangzhou China; ^5^ State Key Laboratory for Diagnosis and Treatment of Infectious Diseases, National Clinical Research Center for Infectious Diseases, Collaborative Innovation Center for Diagnosis and Treatment of Infectious Diseases, The First Affiliated Hospital, School of Medicine Zhejiang University Hangzhou China; ^6^ Shaoxing Tongchuang Biotechnology Co., Ltd Shaoxing China; ^7^ School of Basic Medical Sciences and Forensic Medicine Hangzhou Medical College Hangzhou China

**Keywords:** atrophic gastritis, benzo(a)pyrene, gut microbiota, *Helicobacter pylori*, Mongolian gerbils

## Abstract

Food chemical and microbiological contamination are major global food safety issues. This study investigated the combined effects of the food‐borne pathogen *Helicobacter pylori* (*H. pylori*) and the pollutant benzo(a)pyrene (Bap) on atrophic gastritis and gut microbiota in Mongolian gerbils. The results demonstrated that simultaneous administration of *H. pylori* and Bap caused more severe weight loss, DNA damage, and gastritis in Mongolian gerbils compared with those exposed to *H. pylori* or Bap alone. The combination also significantly increased the serum level of proinflammatory cytokines, including IL‐1β (*p* < .05), IL‐6 (*p* < .0001), and TNF‐α (*p* < .05). Additionally, the *H. pylori* and Bap combination altered the composition of gut microbiota in Mongolian gerbils: the relative abundance of *Lactobacillus* and *Ligilactobacillus* at the genus level (*p* < .05) was significantly reduced while the relative abundance of *Allobaculum* and *Erysipelotrichaceae* enhanced (*p* < .0001, *p* < .05). Our study revealed that the synergy of *H. pylori* and Bap can boost the development of atrophic gastritis and lead to gut microbiota dysbiosis in Mongolian gerbils, which provides essential implications for preventing contaminated foods to sustain life and promote well‐being.

## Graphical abstract

*Helicobacter pylori* (*H. pylori*) infection and benzo(a)pyrene (Bap) exposure are closely linked to food safety and are relatively common in daily life. This study reveals the combined toxic effects of *H. pylori* and Bap in causing atrophic gastritis and gut microbiota dysbiosis in Mongolian gerbils, which provides essential implications for preventing contaminated foods to sustain life and promote well‐being.

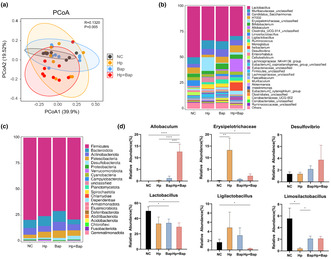

## 1 INTRODUCTION

Food microbiological contamination has increasingly become a significant public health concern worldwide. The global prevalence of atrophic gastritis is about 25% in the study population (Yin et al., 2022). Among various risk factors, *Helicobacter pylori* infection is considered the most frequent cause of atrophic gastritis (Yang & Hu, 2021). Studies have shown that *H. pylori* (a gram‐negative bacterium) infection, mainly caused by oral–oral transmission, is closely related to gastrointestinal diseases including gastritis, gastric and duodenal ulcers, etc. (Bashir & Khan, 2023; Sharndama & Mba, 2022). Previous research has also proven that *H. pylori* infection can lead to a progression from chronic nonatrophic gastritis to atrophic gastritis, followed by intestinal metaplasia and dysplasia, all of which are risk factors for gastric cancer (Hwang et al., 2018). Additionally, *H. pylori* infection is known to alter gut microbiota. Even though the mechanisms are unclear, some research has raised factors that may be involved, such as virulence factors, host immune responses, and modifications to gastric acidity (Tao et al., 2020; Ye et al., 2020).

Food chemical contamination also frequently happens and is another potential risk to gastric health. During the production of smoked and baked foods, a group of harmful organic compounds called polycyclic aromatic hydrocarbons (PAHs) are generated (Bansal & Kim, 2015). Among them, benzo(a)pyrene (Bap) is one of the most characteristic and toxic members and is widely present in water, air, and soil (Bukowska et al., 2022). The major sources of human exposure to Bap are the consumption of smoked, roasted, or fried foods (Ajayi et al., 2016). Thus, the gastric mucosa becomes the first several places to contact with Bap after oral ingestion. Once absorbed into the bloodstream, Bap quickly distributes throughout the body, which can cause DNA damage, gastric inflammation, severe lung diseases, etc. (Dai et al., 2020; Minkina et al., 2020; Zheng et al., 2022).

As *H. pylori* infection and simultaneously food‐borne exposure to Bap is very common in daily life, it is necessary to elucidate their combined effects. In this study, we investigated DNA damage, pathological gastric changes, cytokine production, and gut microbiota in Mongolian gerbils to understand the synergetic effects of *H. pylori* infection and Bap in causing gastritis and intestinal flora dysbiosis. Among various animal models, Mongolian gerbils have a lower natural incidence of gastritis, a longer lifespan, and a better susceptibility to *H. pylori* (Ansari & Yamaoka, 2022a; Yokota et al., 1991), thus serving as a suitable host for stable colonization of *H. pylori* and long‐term observations. The pathological changes in the gastric mucosa of Mongolian gerbils are also analogous to those found in humans (Mishra et al., 2019). Our research provides a reference for further elucidating the synergistic effects of *H. pylori* infection and Bap exposure as well as its potential mechanisms, while also providing a starting point for assisting in the formulation of eradication therapy to prevent physiological injury and gut microbiota disorders.

## 2 MATERIALS AND METHODS

### 2.1 Reagents

*Helicobacter pylori* Sydney strain 1 (SS1), adjusted to a bacterial density of 1 × 10^9^ CFU/mL by turbidity method, was purchased from Hangzhou Zhiyuan Medical Laboratory Co., Ltd. SPF male Mongolian gerbils (*n* = 36) were obtained from the Animal Center of Hangzhou Medical College [Production License No. SCXK (Zhejiang) 2019‐0002, Usage License No. (Zhejiang) 2019‐0011]. The Laboratory Animal Management guidelines were followed during all experiments. Benzo(a)pyrene was purchased from Shanghai Yiboyuan Biotech Co., Ltd. Comet assay kit for DNA damage detection was purchased from Jiangsu KeyGen Biotech Co., Ltd. Bacterial microbiochemical identification tube‐urease was bought from Qingdao Hope Biotech Co., Ltd (China). The Warthin–Starry silver staining solution was purchased from Beijing Leagene Biotechnology Co., Ltd. Formaldehyde and anhydrous ethanol were purchased from Shanghai Lingfeng Chemical Reagent Co., Ltd. Xylene was obtained from Sinopharm company. Mouse IL‐1β ELISA kit, mouse IL‐6 ELISA kit, and mouse TNF‐α ELISA kit were purchased from Proteintech Group, Inc.

### 2.2 Experimental design

**FIGURE 1:**
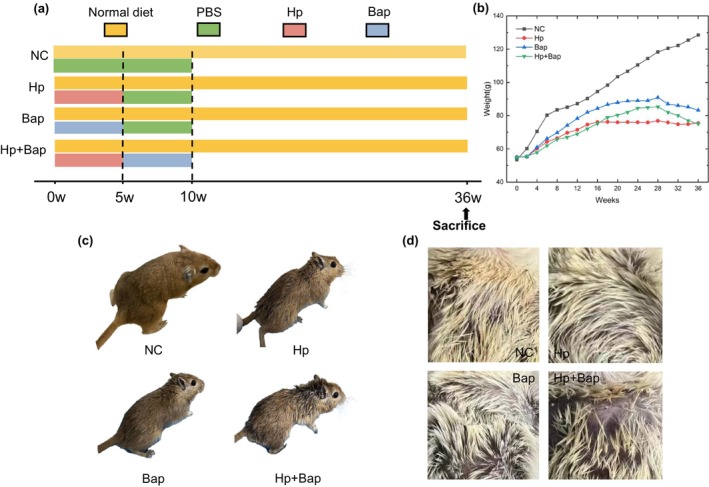
Effects of Hp, Bap, Hp + Bap on Mongolian gerbils. (a) Experimental scheme. (b)Weight changes of each group within 36 weeks. (c) Pictures of representative Mongolian gerbils in week 10. (d) Images of the fur of the same Mongolian gerbils near the neck.

Thirty‐six 2‐month‐old male specific pathogen‐free Mongolian gerbils (50 ± 10 g), purchased from the Animal Center of Hangzhou Medical College, were randomly and blindly divided into four groups (*n* = 9) (Figure 1a): Negative Control (NC) group, *H. pylori* infection (Hp) group, Bap‐induced (Bap) group, *H. pylori*, and Bap co‐treated (Hp + Bap) group. From week 1 to week 5, 0.5 mL of phosphate‐buffered saline (PBS) was given weekly to the NC group by gavage. The Mongolian gerbils in both Hp group and Hp + Bap group received a weekly gavage of 0.5 mL *H. pylori* suspension (Hangzhou Zhiyuan Medical Laboratory Co., Ltd., China) at a concentration of 1 × 10^9^ CFU/mL, while the Mongolian gerbils in Bap group received a weekly gavage of 0.5 mL Bap (Shanghai Yiboyuan Biotech Co., Ltd., China) dissolved in salad oil at a concentration of 5 mg/mL. From week 6 to week 10, the Mongolian gerbils in the NC, Hp, and Bap groups received a weekly gavage of 0.5 mL PBS, while the Mongolian gerbils in the Hp + Bap group received a weekly gavage of 0.5 mL Bap at a concentration of 5 mg/mL. All gerbils fast overnight before each gavage. At week 36, the blood samples from each group were collected for the comet assay and serological assessment of cytokines, while the fecal samples were collected and subjected to 16S rRNA sequencing analysis. On the last day of the experiment, all Mongolian gerbils were sacrificed after fasting for 12 h. Feces, serum, and gastric biopsies were collected and stored at −86°C (Zhongke Meiling Cryogenics Co., Ltd.) for further analysis. *H. pylori* colonization in the stomach was detected by rapid urease test (RUT) and Warthin–Starry staining, and the pathological diagnosis of gastric biopsies was performed by hematoxylin and eosin (H&E) staining. The animal experiments in this study were approved by the Animal Experimental Ethical Committee of Hangzhou Medical College (No. 2021‐115).

### 2.3 Status observation and weight determination

The state of Mongolian gerbils in each group was observed before, during, and after each administration, while their differences in hair, perianal dirt, and kyphosis were recorded. The weight of all the Mongolian gerbils was measured and collected weekly from the beginning to the end of the study.

### 2.4 Determination of *H. pylori* colonization

Rapid urease test (RUT) and Warthin–Starry staining were used to assess the *H. pylori* colonization status. The gastric biopsies of Mongolian gerbils were taken and placed in liquid urease reagent (Qingdao Hope Biotech Co., Ltd., China). If the indicator turned red within 30 min, the RUT was positive. The gastric biopsies were also taken for Warthin–Starry staining, which was fixed with a 10% neutral formalin solution and operated according to the instructions of the Warthin–Starry staining kit (Beijing Leagene Biotechnology Co., Ltd., China). A fluorescence microscope (Eclipse 80i, Nikon, Japan) was used to observe the results.

### 2.5 Histopathological analysis

The gastric biopsies of Mongolian gerbils were fixed with 10% neutral formalin solution, dehydrated by an automatic dehydrator (ASP300S, Leica, Wetzlar, Germany), rendered transparent, soaked in wax, embedded in paraffin, and finally cut into 3‐μm‐thick sections. Subsequently, the sections were stained with H&E solution, sealed with neutral balsam, and eventually observed under a microscope (Olympus Corporation, Japan). According to the Operative Link for Gastritis Assessment (OLGA) grading and staging system (Rugge et al., 2007), the degree of gastritis and atrophy was scored (0–3).

### 2.6 Serological assessment of cytokines

Mouse IL‐1β ELISA kit, mouse IL‐6 ELISA kit, and mouse TNF‐α ELISA kit (Proteintech Group, Inc., Wuhan, China) were used to quantify the corresponding serum level of cytokines (IL‐1β, IL‐6, and TNF‐α) in Mongolian gerbils according to the manufacturer's protocol. The data were analyzed by applying the ELISA Calc regression fitting calculation procedure (Haoke Biotechnology Co., Ltd., Hangzhou, China).

### 2.7 Comet assay

Comet assay, also called single‐cell gel electrophoresis (SCGE), is a versatile and simple method to measure DNA damage in cells (Singh, 2016). We isolated peripheral lymphocytes from Mongolian gerbils in each group and conducted the comet assay using a DNA damage detection kit (Jiangsu KeyGen Biotech Co., Ltd.) according to the manufacturer's protocol. Two hundred cells were randomly selected for measurement in each group under the fluorescence microscope. Then, the CASP analysis software (Beijing Biolaunching Technologies Co., Ltd.) was applied to measure the comet length (total length), tail length, tail moment, Olive tail moment (OTM), and percentage of DNA in the tail (%tail DNA). As a previous study has shown (Anderson et al., 1994), the DNA damage conditions of the cell were divided into five categories corresponding to the %tail DNA: no damage, <5% (grade I); low‐level damage, 5%–20% (grade II); medium‐level damage, 20%–40% (grade III); high‐level damage, 40%–95% (grade IV); complete damage, >95% (grade V). Accordingly, the degree of DNA damage in each experimental group was observed and graded.

### 2.8 Real‐time quantitative PCR

The total RNA of the liver tissue was extracted by a total RNA isolation kit (Haoke Biotechnology Co., Ltd., Hangzhou, China) following the manufacturer's instructions. Then, an All‐in‐One First‐Strand cDNA Synthesis SuperMix for qPCR (TransGen Biotech Co., LTD, Beijing, China) was used immediately to transform RNA into cDNA. The expression level of CYP1A1 mRNA was detected by a Roche qPCR kit. The reaction conditions were as follows: A total of 40 cycles were performed at 94°C for 10 min, 94°C for 15 s, and 60°C for 30 s. Sequences of amplified primers are displayed in Table 1. The relative mRNA levels were analyzed by method 2^−ΔΔCt^.

**TABLE 1:** Sequence of primers.

Gene	Forward primer (5′‐3′)	Reverse primer (5′‐3′)
GAPDH	AACAGCAACTCCCACTCTTCC	TGGTCCAGGGTTTCTTACTCC
CYP1A1	GCCAATGTCATCTGTGCCATAT	CAGGTAACGGAGGACAGGAAT

### 2.9 16S rRNA gene analysis

DNA from different samples was extracted using the CTAB according to the manufacturer's instructions. The V3–V4 variable regions of bacterial 16S rRNA genes were amplified using the upstream primer 341F (5′‐CCTACGGGNGGCWGCAG‐3′) and the downstream primer 805R (5′‐GACTACHVGGGTATCTAATCC‐3′). The PCR products were purified by AMPure XT beads (Beckman Coulter Genomics, Danvers, MA, USA) and quantified by Qubit (Invitrogen, USA). For the double‐ended data obtained by sequencing, the data needed to be first split according to barcode information. Then, data splicing and filtering were performed by software including Cutadapt (v1.9), FLASH (v1.2.8), Fqtrim (v0.94), and Vsearch (v2.3.4). Through qiime dada2 denoise‐paired, DADA2 was used for length filtering and dereplication, after which ASV feature sequences and abundance tables were obtained. The α‐diversity analysis and β‐diversity analysis were conducted on these results. The LEfSe (linear discriminant analysis effect size) analysis was performed using the Omicstudio platform and the analysis result was estimated by LDA (linear discriminant analysis) score.

### 2.10 Data analysis

To analyze the data, we used GraphPad Prism 9.5.1 (GraphPad Software Inc., San Diego, CA, USA). Mann–Whitney *U* test comparison was used to test the differences between two groups, while one‐way ANOVA or the Kruskal–Wallis test was utilized to test significant differences among multiple groups. GraphPad Prism 9.5.1, Origin64, and R software were employed to prepare the figures. *p* < .05 was considered statistically significant. The data are shown as mean ± SEM, *n* = 9.

## 3 RESULTS AND DISCUSSION

### 3.1 Effects of *H. pylori* and Bap on Mongolian gerbils' growth

To examine the potential synergistic effects of *H. pylori* and Bap on Mongolian gerbils, except the NC group, Mongolian gerbils in groups were orally administered with Hp, Bap, and Hp + Bap, separately. The gerbils' body weight was recorded weekly over 36 weeks (Figure 1b). Compared with the NC group, we found that the body weight of Mongolian gerbils in the Hp, Bap, and Hp + Bap groups decreased, indicating that all treatments negatively affected growth. Notably, the Hp + Bap group exhibited the lowest body weight up to week 16. After week 16, the body weight of the Hp group remained virtually unchanged, which might be attributed to the colonization of *H. pylori* and the development of chronic gastritis (Xi et al., 2023). However, the weight of Mongolian gerbils in the Hp + Bap group slightly increased after 16 weeks and higher in the Hp group, which appears contradictory to our initial findings. Although the mechanisms are still unknown, we conjectured that the toxicity of Bap may have reduced the *H. pylori* population in Mongolian gerbils, leading to less severe gastritis compared to the Hp group. Additionally, hormesis may also be responsible for this outcome (Davies, 2016). Hormesis is an adaptive response of organisms, that is, toxic agents themselves or combined with another substance can stimulate transient adaptation (Li et al., 2019). Interestingly, the weight of Mongolian gerbils in the Hp + Bap group decreased rapidly after 28 weeks, which might be caused by the increasingly severe gastritis due to the synergistic effects of *H. pylori* and Bap.

The physical conditions of Mongolian gerbils were also observed and recorded weekly (Figure 1c,d). Mongolian gerbils in the NC group consistently exhibited thick, shiny fur, normal posture, and no perianal filth. In contrast, Mongolian gerbils manifested symptoms such as alopecia, kyphosis, and diarrhea after a long time feeding with Hp + Bap. The Hp group showed signs of messy, dull fur without perianal filth, while the Bap group exhibited similar symptoms of hair loss and dull fur without perianal filth. Previous research has proved that Bap can accelerate alopecia areata in B6/129 F1 mice (Balansky et al., 2006), which is in line with our results. *H. pylori* infection also appears to be more common in patients with alopecia areata (Lee et al., 2019). However, further research is necessary to clarify the connections between these symptoms and atrophic gastritis caused by Hp + Bap.

### 3.2 The synergistic effects of *H. pylori* and Bap on aggravating peripheral lymphocytes DNA damage in Mongolian gerbils

**FIGURE 2:**
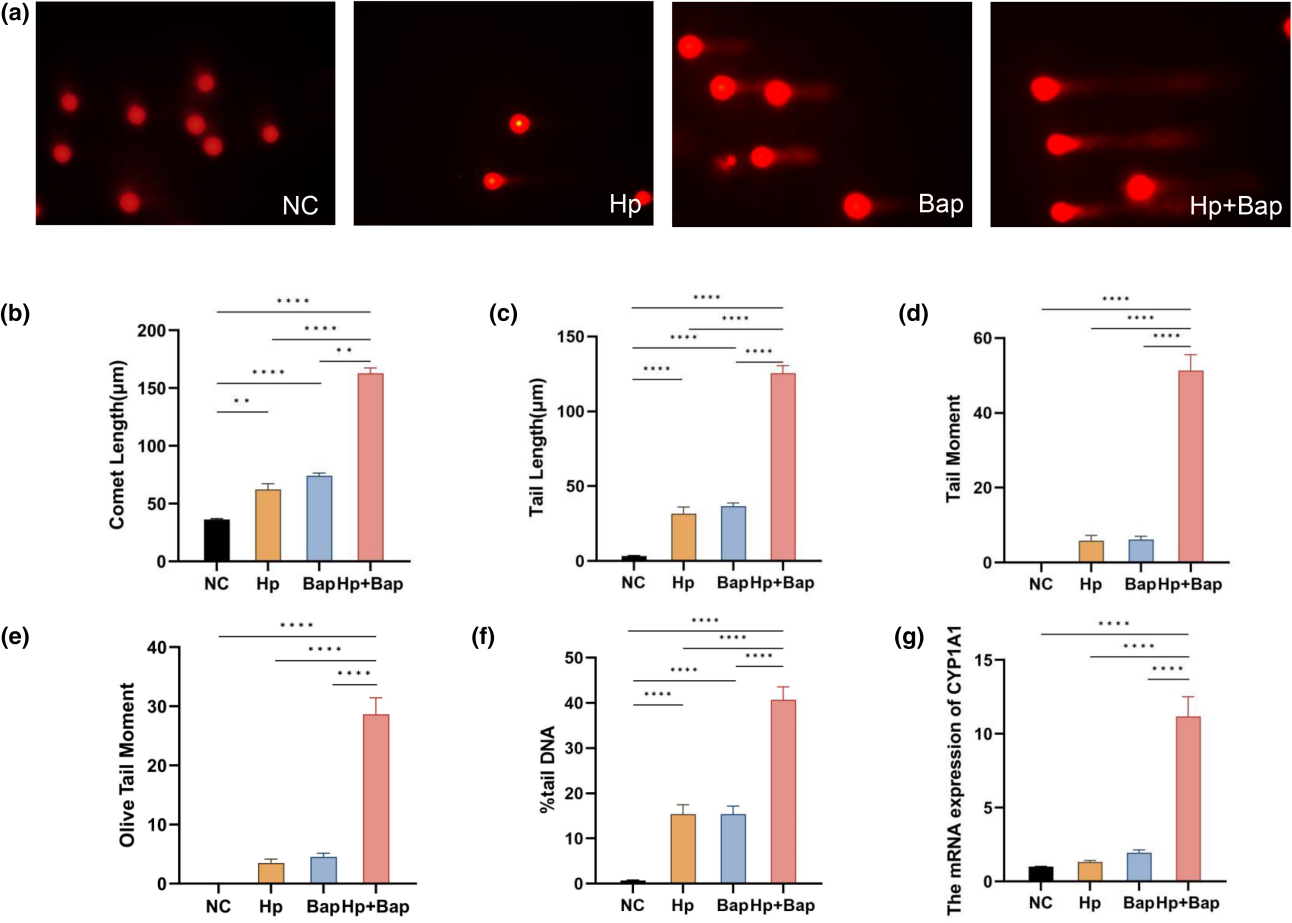
Peripheral lymphocytes DNA damage of Mongolian gerbils in each group. (a) Images of peripheral lymphocytes in different groups under a fluorescence microscope. Bar chart reporting comet length (b), tail length (c), tail moment (d), Olive tail moment (e), and %tail DNA (f) of the damaged cells in each group. (g) The mRNA expression levels of CYP1A1 in liver tissue of Mongolian gerbils. The data were collected using CASP analysis software. Data are presented as mean ± SEM, *n* = 9. **p < .01, ****p < .0001.

In the nucleus, DNA is arranged in loops and attached to the nuclear matrix (Anachkova et al., 2005). The mechanism of the comet assay is that when DNA strand breaks exist, it results in the relaxation of DNA supercoiling, causing exposure of negative charges (Collins et al., 2023). Under relaxation, DNA loops that are still connected to the nuclear matrix are pulled to the anode by an electric field. This creates a distinctive “comet tail” that can be observed under a fluorescence microscope. The amount of DNA present in the tail indicates the frequency of breaks. Fluorescence imaging (Figure 2a) showed that cells in the NC group maintained round shapes with well‐defined nuclear boundaries. In contrast, the characteristic “comet tail” appeared in the Hp + Bap group, indicative of broken DNA fragments migrating out of the nucleus. Mongolian gerbil cells in the Hp group and Bap group also exhibited “comet tails,” albeit shorter than those in the Hp + Bap group. Figure 2f shows that the %tail DNA in the Hp + Bap group was significantly higher than that in the NC, Hp, and Bap groups (*p* < .0001), which was recommended by the OECD as the best descriptor for the break of DNA (Collins et al., 2023). Similar patterns were seen in the comet length (Figure 2b), tail length (Figure 2c), tail moment (Figure 2d), and OTM (Figure 2e) in the Hp + Bap group compared to other groups (*p* < .0001). Besides, both the Hp and Bap groups exhibited higher %tail DNA than the NC group (*p* < .0001). Notably, the %tail DNA of the Hp + Bap group exceeded 40%, indicating a high level of damage (grade III), while the %tail DNA of both the Hp and Bap groups ranged from 5% to 20%, representing a comparatively low level of damage (grade I) (Table 2). All these results indicated the synergistic effects of *H. pylori* and Bap on aggravating DNA damage in Mongolian gerbils' peripheral lymphocytes.

**TABLE 2:** Peripheral lymphocytes DNA damage of Mongolian gerbils in each group.

	Comet length	Tail length	Tail moment	Olive tail moment (OTM)	%tail DNA
NC	36.20 ± 2.98^c^	3.32 ± 1.84^c^	0.03 ± 0.03^b^	0.11 ± 0.10^b^	0.67 ± 0.64^c^
Hp	62.31 ± 22.08^b^	31.64 ± 19.87^b^	5.86 ± 6.26^b^	3.52 ± 2.88^b^	15.39 ± 9.39^b^
Bap	74.12 ± 10.27^b^	36.54 ± 10.35^b^	6.10 ± 4.07^b^	4.54 ± 2.67^b^	15.35 ± 8.19^b^
Hp + Bap	162.79 ± 20.99^a^	125.61 ± 21.90^a^	51.33 ± 18.97^a^	28.65 ± 12.55^a^	40.67 ± 12.91^a^

*Note*: Values represent the mean ± SD, *n* = 9. Values with superscript letters a, b, and c are significantly different across columns (*p* < .05).

According to previous research, *H. pylori* infection induces chronic inflammation and generates specific bacterial virulence factors, leading to the accumulation of reactive oxygen species (ROS) and oxidative stress, resulting in DNA damage in host cells (Salvatori et al., 2023). DNA damage plays a crucial role in the progression from gastritis to gastric cancer after *H. pylori* infection (Kalisperati et al., 2017). In clinical trials, eradication therapy as an effective treatment has been demonstrated to significantly reduce DNA damage and oxidative stress in peripheral lymphocytes, and may also help prevent the development of gastric cancer in *H. pylori*‐positive patients (Dulger et al., 2011). Additionally, studies have shown that Bap can also induce oxidative stress through its impact on ROS formation (Gao et al., 2022). Therefore, we hypothesize that the mechanism of the combined effect of *H. pylori* and Bap inducing more severe DNA damage is associated with their synergistic effects on oxidative stress.

The human cytochrome P450 (CYP) 1A1 gene, part of the CYP1 family, is primarily expressed in the liver, lungs, and intestines (Klomp et al., 2020; Stading et al., 2021). As shown in Figure 2g, the expression of the CYP1A1 gene in the liver tissue of Mongolian gerbils in the Hp + Bap group was significantly higher than in the Hp and Bap groups (*p* < .0001). The mechanism by which Hp infection elevates CYP1A1 gene expression is not well documented. However, Bap is known to bind to the aryl hydrocarbon receptor (AhR) after exposure, resulting in the nuclear translocation of AhR which induces transcription and expression of downstream genes, such as CYP1A1 (Mescher & Haarmann‐Stemmann, 2018). CYP1A1 mainly catalyzes the metabolism of Bap to form BPDE‐DNA adducts, which are closely associated with mutations (Reed et al., 2018). BPDE‐DNA adducts tend to cause guanine to thymine base transversion, thereby promoting cellular mutations, DNA damage, inflammatory cytokine expression, and potentially leading to inflammation and even carcinogenesis (Willis et al., 2018). Our study demonstrates that the combination of *H. pylori* and Bap significantly induces CYP1A1 gene expression in Mongolian gerbils compared to *H. pylori* or Bap alone, partially elucidating the molecular mechanism behind the exacerbated gastritis and toxic damage observed. These findings emphasize the necessity for further studies to understand the interplay between *H. pylori* and Bap in order to develop effective interventions.

### 3.3 Combined administration of *H. pylori* and Bap caused more severe gastritis in Mongolian gerbils

**FIGURE 3:**
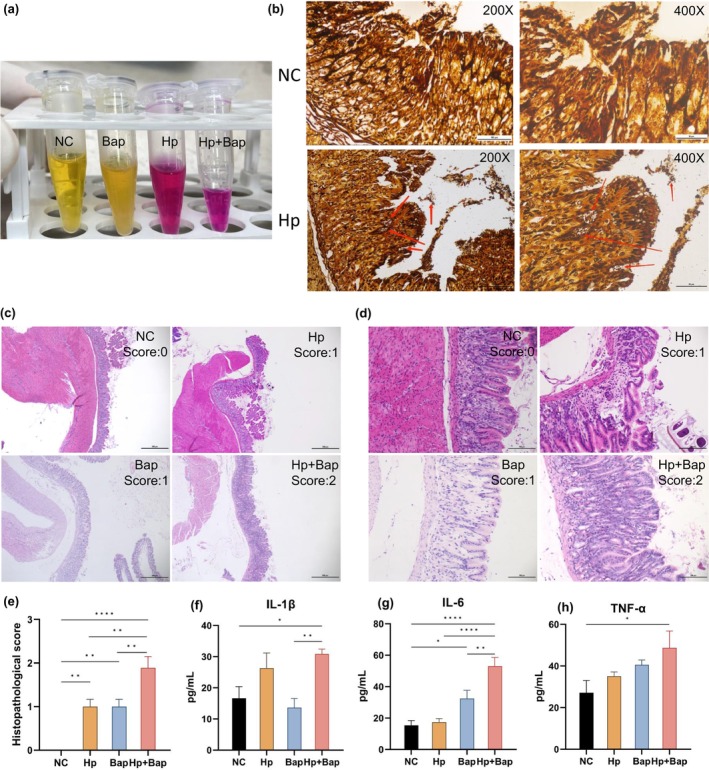
*Helicobacter pylori* colonization and gastric tissue injury. (a) The results of the Rapid urease test (RUT). (b) Representative images of gastric biopsies after Warthin–Starry staining in the NC and Hp + Bap groups. (c) Representative images of gastric biopsies after H&E staining, HE × 40. (d)The same biopsies as the images above, HE × 200. (e) Histopathological scores (0–3) of the gastric antrum biopsies based on H&E staining. The serum level of IL‐1β (f), IL‐6 (g), and TNF‐α (h) among the four groups. Data are shown as mean ± SEM, *n* = 9. *p < .05, **p < .01, and ****p < .0001.

Rapid urease test (RUT) and Warthin–Starry staining were performed to assess the colonization of *H. pylori*. In the 36th week of the experiment, all Mongolian gerbils in the four groups were sacrificed for *H. pylori* infection detection. In the RUT, the color shifted from yellow to purple in both the Hp and Hp + Bap groups, indicating positive results. Conversely, the color remained unchanged (yellow) in the NC and Bap groups, suggesting the absence of *H. pylori* colonization (Figure 3a). Histological examination via Warthin–Starry, a common diagnostic standard for *H. pylori* infection (Ansari & Yamaoka, 2022b), revealed typical short rod‐shaped or seagull‐shaped black particles on the surface of the gastric mucosa and within the glandular lumen in the Hp and Hp + Bap groups (Figure 3b), confirming *H. pylori* colonization. No *H. pylori* colonization was detected in the gastric biopsies of the NC and Bap groups. The results of the RUT and Warthin–Starry staining verified that *H. pylori* SS1 colonized the stomach of Mongolian gerbils in the Hp and Hp + Bap groups at a rate of 100%. Previous research suggests that motility, urease production, and adhesion are key mechanisms for *H. pylori* colonization (Malfertheiner et al., 2023). From an immunological perspective, *H. pylori* sustains a dynamic equilibrium of pro‐inflammatory and anti‐inflammatory cytokines via intricate multilateral crosstalk between the gastric epithelium and immune responses, facilitating persistent colonization (Yang & Hu, 2022).

The results of the histopathological analysis showed that all the animals developed atrophic gastritis in the Hp + Bap group (Figure 3c,d). The histopathology score system demonstrated significantly higher scores in the Hp + Bap group compared to the Hp or Bap group (*p* < .01) (Figure 3e). Some damage was also observed in the Hp and Bap groups, although not severe enough to result in atrophic gastritis. Besides, all the Mongolian gerbils in the Hp + Bap group were considered to have atrophic gastritis after histopathological examination, including atrophy of gastric glands, reduced quantity, and inflammatory cell infiltration (Crafa et al., 2018). Notably, atrophic gastritis in the Mongolian gerbils did not progress to gastric cancer within the study period, which may be attributed to the short observation duration (Cortes‐Marquez et al., 2018).

The serum level of proinflammatory cytokines, including IL‐1β, IL‐6, and TNF‐α in each group may also serve as supporting evidence (Figure 3f,g,h). Compared with the NC group and Bap group, the Hp + Bap group showed a significant increase in the expression level of IL‐1β in serum (*p* < .05). As for IL‐6, the expression level in the Hp + Bap group was markedly higher than that in the Hp group (*p* < .0001) and Bap group (*p* < .01). Moreover, the levels of TNF‐α in the Hp + Bap group also showed an upregulation compared with the NC group (*p* < .05). According to previous research, cytokines can chemotactically attract and activate neutrophils, leading to nonspecific inflammatory responses in the body and causing damage to the gastric and duodenal mucosa (Costa et al., 2009). The elevation of TNF‐α has been shown to be one of the indicators of mice with atrophic gastritis (Tong et al., 2021). It acts as an upregulator of the nitric‐oxide‐dependent pathway in carcinogenesis, which can hinder DNA repair and cause DNA damage according to studies (Bagheri et al., 2018). This is consistent with the above results obtained from our DNA damage experiment. IL‐1β promotes inflammation and the release of inflammatory mediators such as IL‐2, IL‐6, and TNF‐α (Dinca et al., 2022). It also shows a positive correlation with *H. pylori* colonization, thereby aggravating gastric inflammation (Moradipour et al., 2018). During gastritis, IL‐6 acts as a defense‐related pro‐inflammatory cytokine (Santos et al., 2021), but can lead to chronic inflammatory diseases and cytokine storms when produced excessively (Hirano, 2021). In terms of gastric cancer, it has been reported that IL‐6 can lead to an abnormal immune response by the JAK/STAT signaling pathway, which underlies the development of gastric cancer (Dinca et al., 2022).

Our results showed that the expression levels of TNF‐α, IL‐1β, and IL‐6 in the Hp + Bap group were significantly elevated compared to other groups, supporting that the combined effects of *H. pylori* and Bap caused more severe gastritis in Mongolian gerbils in some extent. This result is consistent with the above results of status observation and histopathological analysis.

### 3.4 Effects of *H. pylori* and Bap combination on gut microbiota composition in Mongolian gerbils

**FIGURE 4:**
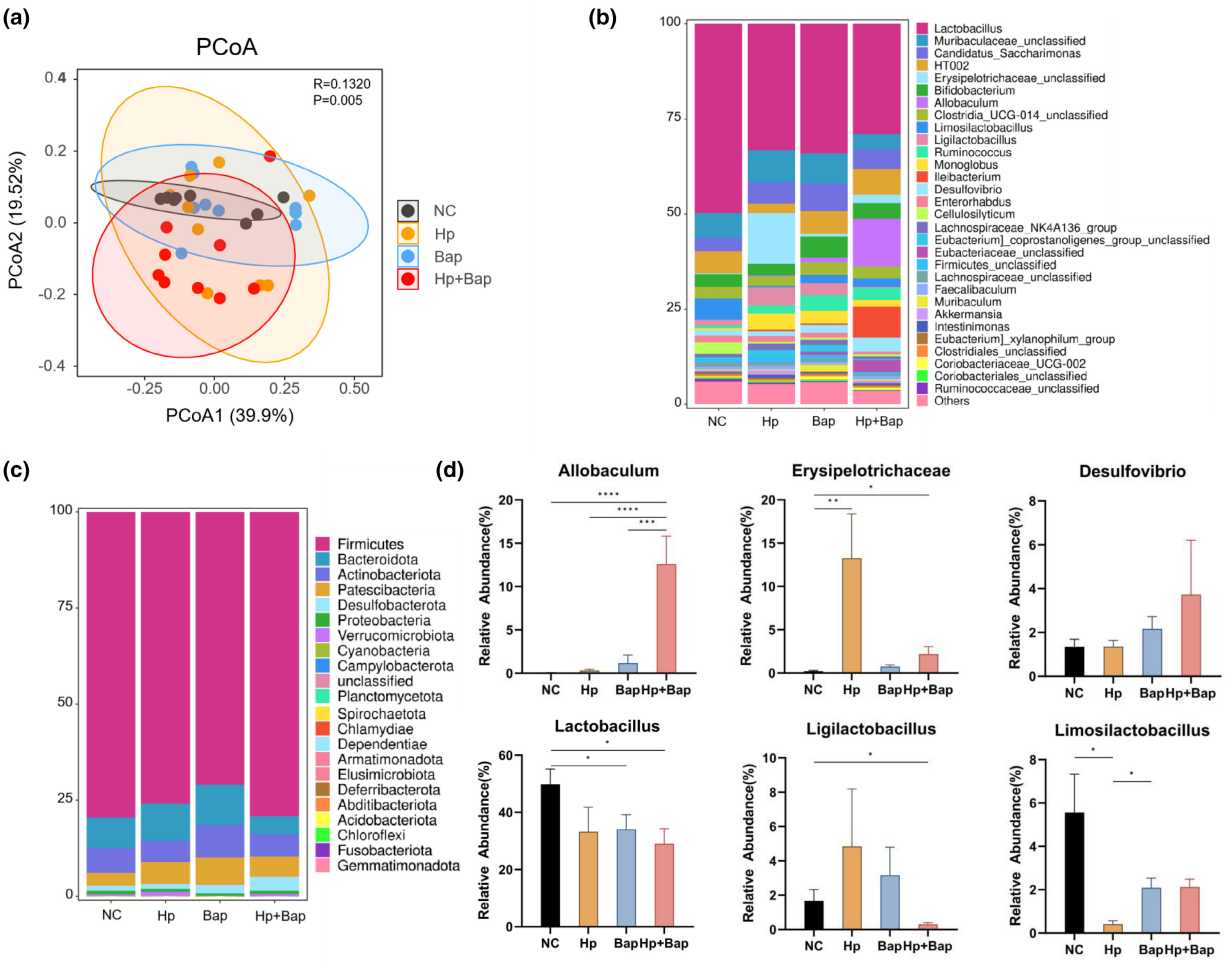
Combination of *Helicobacter pylori* and Bap aggravated gut microbiota dysbiosis in Mongolian gerbils. (a) β‐diversity of the gut microbiota by weighted PCoA plots. Relative abundance of taxa at the phylum (c) and genus (b and d) levels. Data are presented as mean ± SEM, *n* = 9. *p < .05, **p < .01, ***p < .001, and ****p < .0001.

Many gastrointestinal diseases, such as gastritis, are closely related to the gut microbiota (Sharma et al., 2023). Previous studies have investigated the impact of *H. pylori* infection and atrophic gastritis on the intestinal flora (Iino et al., 2020). To explore the changes in the gut microbiota after administration (NC, Hp, Bap, Hp + Bap), we used 16S rRNA gene sequencing to analyze the colonic content samples. β‐diversity was assessed by utilizing weighted principal coordinates analysis (PCoA), indicating a significant difference among the four groups (*p* = .005, Figure 4a). The top 30 most abundant taxa at the phylum and the top 20 most abundant taxa at the genus level are shown in Figure 4c,b. At the phylum level, the abundance of Bacteroidota in the Hp + Bap group decreased significantly (*p* < .05) compared to the Bap group. The abundance of Patescibacteria in the Hp group, Bap group, and Hp + Bap group were increased compared to the NC group, but not significantly. At the genus level (Figure 4d), the combined effects of *H. pylori* infection and Bap exposure resulted in a significant decrease in the relative abundance of *Lactobacillus* and *Ligilactobacillus* (*p* < .05), both of which were the lowest among the four groups. Compared with the NC group, the abundance of *Limosilactobacillus* in the Hp group decreased significantly (*p* < .05), while a similar trend was seen in the Bap group and Hp + Bap group. *Lactobacillus*, *Ligilactobacillus*, and *Limosilactobacillus* are all potential probiotics and have been widely used in human health (Abuqwider et al., 2022; Huang et al., 2022; Rodriguez‐Sojo et al., 2021). Some strains from *Lactobacillus* and *Limosilactobacillus* have been proven to inhibit *H. pylori* infection and tend to ameliorate gastritis in mice (Zhao et al., 2022; Zhou et al., 2022). Taking *Ligilactobacillus* orally has therapeutic effects on inflammatory bowel diseases since they are capable of reestablishing gut microbiota balance as well as restoring gut barrier integrity (Yao et al., 2021). Therefore, the significant decline in the relative abundance of *Lactobacillus*, *Ligilactobacillus*, and *Limosilactobacillus* may lead to gut microbiota dysbiosis, and promote gastrointestinal inflammation and other diseases. On the other hand, the relative abundance of some opportunistic pathogens increased remarkably in the gut of Mongolian gerbils in the treated groups. Compared to the NC group, the Hp + Bap group had a significantly higher relative abundance of *Allobaculum* and *Erysipelotrichaceae* (*p* < .0001, *p* < .05), while the richness of *Allobaculum* in the Hp + Bap group was also higher than that in Hp and Bap groups (*p* < .0001, *p* < .001). Combined administration of Hp + Bap tended to increase the abundance of *Desulfovibrio* as well, but there was no significant difference. According to previous studies, *Allobaculum mucolyticum* plays an important role in the development of intestinal inflammation (van Muijlwijk et al., 2021), since it may secrete quantities of mucin o‐glycans that act on carbohydrate‐active enzymes, which have potent mucolytic capabilities and facilitate bacterial colonization. *Erysipelotrichaceae* are reported to be potential pathogens (Kaakoush, 2015) that overgrow in the gastric microbiota of *H. pylori*‐infected mice and are closely related to gut microbiota in colorectal cancer (Gootenberg et al., 2017). *Desulfovibrio*, an opportunistic pathogen that produces hydrogen sulfide (H_2_S) in the gut, can cause DNA damage, enhance the inflammatory response of the colonic mucosa, and even contribute to colorectal cancer (Nie et al., 2022; Szabo et al., 2013).

**FIGURE 5:**
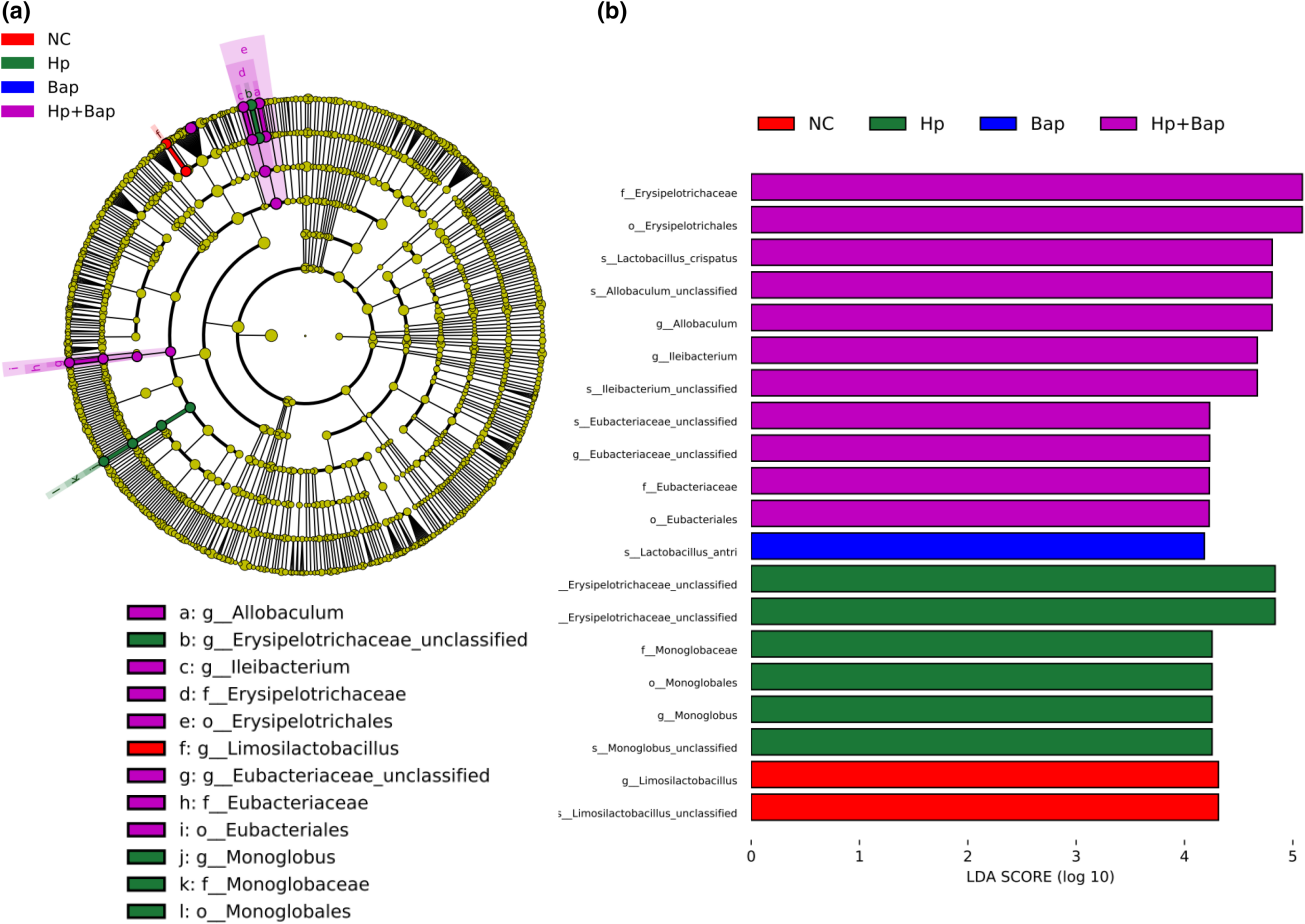
Hp, Bap, and Hp + Bap reshaped the gut microbiota in Mongolian gerbils. (a) LEfSe cladogram demonstrating the taxa enriched in the NC group (red), Hp group (green), Bap group (blue), and Hp + Bap group (purple). (b) Discriminative biomarkers between the NC group (red), Hp group (green), Bap group (blue), and Hp + Bap group (purple) are listed. Statistical significance reflects both *p* < .05 for Kruskal–Wallis tests and a logarithmic LDA score >3.0.

Since the discriminant analysis did not identify the predominant taxon, LEfSe analysis was used to distinguish the most significant differences in fecal microbiome composition among the four groups (Figure 5a,b). Several bacteria that are closely related to either intestinal inflammation or colorectal cancer (CRC) including *Allobaculum*, *Erysipelotrichaceae*, and *Ileibacterium* were all significantly overrepresented in the feces of Mongolian gerbils in the Hp + Bap group. Recent studies have demonstrated that *Ileibacterium* is implicated in obesity and intestinal inflammation, while the enrichment of *Ileibacterium Valens* may also promote carcinogenesis (Fu et al., 2023; Lin et al., 2022).

Our results suggest that the combined effects of *H. pylori* and Bap caused a more significant gut microbiota imbalance in the Mongolian gerbils than either *H. pylori* or Bap alone.

There are some limitations in our study. Firstly, the Mongolian gerbil animal model cannot completely reproduce atrophic gastritis in humans, thus further clinical trials in human cohorts are urgently needed. Secondly, our research only reveals the synergistic effects of *H. pylori* and Bap on altering gut microbiota homeostasis in Mongolian gerbils, while the precise mechanism still requires further investigation.

## 4 CONCLUSIONS

In summary, we conclude that *H. pylori* and Bap have synergistic toxic effects in causing weight loss, DNA damage, atrophic gastritis, elevated proinflammatory cytokine levels, and gut microbiota dysbiosis in Mongolian gerbils. The potential detrimental impact of Bap exposure in *H. pylori*‐infected patients was raised in this study. Future studies can focus on investigating their mechanisms in inducing this gastric disease in detail and providing an effective strategy to prevent microbiological and chemical food contamination in associated foods.

## AUTHOR CONTRIBUTIONS

**Yilun Huang:** Conceptualization (equal); formal analysis (equal); investigation (equal); visualization (equal); writing – original draft (equal). **Yunxiang Chen:** Investigation (equal); methodology (equal). **Lingfei Ma:** Investigation (equal). **Honggang Guo:** Funding acquisition (equal); investigation (equal). **Hao Chen:** Investigation (equal). **Bo Qiu:** Formal analysis (equal). **Mingfei Yao:** Writing – original draft (equal); writing – review and editing (equal). **Weixin Huang:** Funding acquisition (equal); resources (equal). **Lian Zhu:** Conceptualization (equal); funding acquisition (equal); investigation (equal); methodology (equal); project administration (equal); resources (equal); supervision (equal); validation (equal); writing – review and editing (equal).

## FUNDING INFORMATION

This research was supported by the Basic Public Welfare Research Program of Zhejiang Province (Nos. LGD22H160012, LGD22C040024) and the Medical Science and Technology Project of Zhejiang Province (Nos. 2022KY730, 2023ZL358).

## CONFLICT OF INTEREST STATEMENT

The authors declare no conflict of interest.

## ETHICAL APPROVAL

The animal experiments in this study were approved by the Animal Experimental Ethical Committee of Hangzhou Medical College (No. 2021‐115).

## INFORMED CONSENT

Written informed consent was obtained from all study participants.

## Data Availability

The data that support the findings of this study are available from the corresponding author upon reasonable request.
